# Perceptions of pediatric neurologists on the ideal candidate: a nationwide survey

**DOI:** 10.1186/s12909-026-08938-w

**Published:** 2026-03-05

**Authors:** Ahmed Bamaga, Hebah Qashqari, Anas Alyazidi, Mohamed O. E. Babiker, Jihan Madani, Montaha Almudhry, Sulaiman Almobarak, Fatima Ismail

**Affiliations:** 1 Department of Pediatrics, Faculty of Medicine, King Abdulaziz University, Jeddah, Saudi Arabia; 2 Department of Pediatrics, King Faisal Specialist Hospital & Research Centre, Jeddah, Saudi Arabia; 3 Neuroscience Center, Al Jalila Children’s Specialty Hospital, Dubai, United Arab Emirates; 4 Department of Pediatrics, Faculty of Medicine, Mohammed Bin Rashed University of Medicine and Health Sciences, Dubai, United Arab Emirates; 5 Neuroimmunology, Neuroimmunology and Headache Department, Neuroscience center, King Faisal Specialist Hospital & Research Center, Riyadh, Saudi Arabia; 6 College of Medicine, Alfaisal University, Riyadh, Saudi Arabia; 7 Section of Neurosciences, Department of Pediatric Neurology, King Fahad Specialist Hospital, Dammam, Saudi Arabia; 8 Pediatric Neurology Department, King Saud Medical City, Riyadh, Saudi Arabia; 9 Pediatric Department, College of Medicine and Health Sciences, United Arab Emirates University, Al Ain, United Arab Emirates

**Keywords:** Pediatric neurology, Internship and residency, Program development, Education, Medical, Saudi Arabia

## Abstract

**Background:**

Pediatric neurology residency selection is increasingly holistic globally, yet the specific attributes prioritized by program directors (PDs) in Saudi Arabia and the United Arab of Emirates (UAE), where Saudi Commission for Health Specialties (SCFHS)-accredited programs are established, remain unexamined. This study investigates the perceptions of PDs, compared to non-PD pediatric neurologists, regarding the ideal candidate.

**Methods:**

A multicenter, cross-sectional survey was distributed to pediatric neurology physicians across Saudi Arabia and the UAE, including 9 PDs and 34 non-PD faculty. The mixed-methods survey assessed the importance of academic credentials, non-cognitive attributes, and other qualifications using Likert scales, ranking exercises, and free-text responses. Data were analyzed using descriptive statistics, multivariable logistic regression, and thematic analysis of free-text responses.

**Results:**

Quantitative analysis of attribute rankings identified professionalism/attitude (69.8%) and clinical rotation performance (58.1%) as the most frequently cited critical factors. Thematic analysis of open-ended responses further elucidated these priorities and provided rich contextual detail on the ideal candidate profile. Quantitative ratings confirmed that both groups prioritized these non-cognitive and clinical attributes over academic metrics. However, significant differences emerged: PDs rated communication skills substantially higher than non-PDs (mean 5.0 vs. 4.5, *p*=0.03) and also assigned greater importance to interview performance (mean 4.7 vs. 3.9, *p*=0.04). Multivariable analysis confirmed that valuing communication skills (aOR=5.81; 95% CI: 1.18–28.6; *p*=0.03) and interview performance (aOR=2.45; 95% CI: 1.02–5.88; *p*=0.045) were independent predictors of being a PD.

**Conclusion:**

While the pediatric neurology community broadly endorses a holistic selection model, PDs in Saudi Arabia and the UAE distinctly prioritize superior communication skills and interview performance. These findings suggest that refining selection processes to effectively evaluate these competencies is essential for aligning recruitment with the priorities of those overseeing training.

**Supplementary Information:**

The online version contains supplementary material available at 10.1186/s12909-026-08938-w.

## Background

Pediatric neurology has emerged as a critical specialty at the crossroads of rapid scientific discovery and the growing recognition of neurological disorders as a leading cause of morbidity and long-term disability in children worldwide [1]. Globally, the second part of the 20th century saw the establishment of the first training programs in child neurology. Over time, program oversight, curriculum, and resident characteristics have changed [2]. Following this international development, the field in Saudi Arabia has experienced parallel growth, shaped by the establishment and expansion of structured residency programs under the Saudi Commission for Health Specialties (SCFHS) [3]. These five-year training pathways are designed not only to develop clinical competence but also to foster research capability, professionalism, and leadership skills, aligning with the country’s broader healthcare transformation agenda [3–5].

Admission into pediatric neurology residency programs is highly competitive [2]. Applicants are expected to demonstrate strong academic foundations, including accredited medical education, successful completion of internship, licensing examinations, and evidence of scholarly potential [6]. Yet, beyond academic metrics, program directors (PDs) increasingly emphasize the importance of non-cognitive attributes such as professionalism, resilience, communication, adaptability, and teamwork, qualities that often determine success in the demanding and evolving clinical environment of pediatric neurology [7]. International studies across medical specialties consistently highlight this holistic approach to trainee selection, where objective scores may open the door, but personal and professional attributes secure the seat [8, 9].

Despite the global consensus on the value of holistic evaluation, there remains a notable gap in understanding which qualities Saudi pediatric neurology PDs specifically prioritize when identifying their ideal candidate. Such insights are not only academically relevant but also essential for guiding undergraduate medical education, refining mentorship strategies, and informing residency recruitment policies within the local healthcare context. Furthermore, as PDs hold the ultimate responsibility for candidate selection and ranking, this study sought specifically to identify whether their prioritization of candidate attributes differed from the broader faculty perspective.

This study therefore seeks to systematically investigate the perceptions of pediatric neurology residency PDs in Saudi Arabia and the UAE—the nations hosting SCFHS-accredited training programs—regarding the attributes of the ideal residency candidate. By clarifying these expectations, the study aims to provide practical guidance to aspiring trainees, contribute to evidence-based improvements in selection practices, and ultimately support the continued development of a skilled and well-prepared pediatric neurology workforce.

## Materials and methods

### Study design and conceptual framework

Following the Strengthening the Reporting of Observational Studies in Epidemiology (STROBE) guideline [10], this study employed a cross-sectional, convergent parallel mixed-methods design. This approach was chosen to provide a comprehensive understanding of the research problem by collecting and analyzing quantitative and qualitative data simultaneously, then merging the results for interpretation. The work was guided by a pragmatist paradigm, which prioritizes the research question over commitment to a single philosophical worldview and justifies the use of both quantitative (to measure prevalence and relationships) and qualitative (to explore meaning and context) methods. This framework is appropriate for applied health professions education research where the goal is to generate actionable insights. All study procedures were conducted in accordance with the Declaration of Helsinki and its subsequent amendments, as well as Good Clinical Practice (GCP) guidelines. Participation was entirely voluntary, and informed consent was obtained from all study participants.

### Study setting and population

A census approach was used for PDs, with invitations sent to all 12 SCFHS‑accredited pediatric neurology program directors in Saudi Arabia and the United Arab Emirates (UAE) identified via SCFHS records and professional networks. Non‑PD faculty—including former PDs, specialists, and consultants—were recruited via convenience sampling through professional channels, society mailing lists, and open forums. The non‑PD survey was distributed to an estimated pool of 120–150 pediatric neurologists practicing in the region; a precise denominator is unavailable due to the open distribution method.

Therefore, a total of 43 physicians participated. Of the 12 accredited PD positions, one program was in a center still undergoing accreditation at the time of the survey, and one PD was on leave and unavailable. Consequently, 9 of 10 eligible and available PDs completed the survey—a 90% participation rate among accessible PDs, representing 75% of all accredited PD positions. Additionally, 34 non‑PD faculty members participated; a formal response rate for this group cannot be calculated due to the open recruitment method.

This comparative design was implemented to examine whether the priorities of PDs—who hold final selection authority—differ from those of the wider faculty community. Given the census approach for PDs and the exploratory, descriptive aim of comparing group perspectives, a formal power calculation was not performed. The goal was to capture the highest possible response from the finite PD population and a representative convenience sample of non‑PDs.

### Survey development

The survey instrument was developed following a review of international literature on residency selection criteria across medical specialties, complemented by expert input from pediatric neurology faculty in Saudi Arabia and the UAE [6, 11–13]. The instrument was explicitly designed for convergent data collection, pairing Likert-scale items (quantitative) with open-ended, free-text prompts (qualitative) within the same domains (e.g., following a question rating the importance of ‘communication skills,’ a free-text question asked for the top qualities of an ideal resident). This allowed for direct comparison and integration of numerical trends with thematic insights during analysis. The questionnaire included sections on demographic data (such as years in practice, region, and program size), the structure of the selection process, and the perceived importance of various academic criteria (for example, Grade Point Average [GPA], licensing examination scores, and clerkship evaluations). Additional domains covered non-cognitive attributes such as professionalism, resilience, communication skills, adaptability, and teamwork, as well as the role of research experience, leadership qualities, and advocacy (Supplementary Files-1).

### Pilot testing

Prior to dissemination, the draft survey underwent pilot testing with two pediatric neurologists who were not PDs and were not part of the expert panel. The pilot focused on usability, clarity of instructions, item interpretation, and estimated completion time, a process aligned with cognitive interview principles to identify problematic wording or concepts. Formal, verbatim cognitive interviews were not conducted. Feedback from this pilot phase was used to refine wording and improve the logical flow of the questionnaire.

### Data collection

The final version was distributed electronically via a secure online platform (Google Forms^®^). Invitations were sent through official SCFHS communication channels and direct email contact. To optimize the response rate, two reminders were sent at two-week intervals. Data collection was carried out during the period of September and December 2025.

### Data analysis

Data analysis included both quantitative and qualitative approaches. Descriptive statistics, including means, medians, frequencies, and percentages, were used to summarize the characteristics of respondents and their survey responses. Ranking and Likert-scale data were further analyzed to identify the attributes most and least valued by PDs. For comparisons between two independent groups (PDs vs. non-PDs), the Mann-Whitney U test was applied to continuous and ordinal variables, including mean importance scores for candidate attributes and satisfaction ratings. For comparisons involving more than two groups (e.g., institution type, training background categories), the Kruskal-Wallis test was used. All statistical tests were two-tailed with significance set at *p* < 0.05. Free-text responses to the question on the single most important selection factor were reviewed and categorized by the research team, and the frequency of each mentioned attribute was calculated and reported descriptively. They were analyzed using reflexive thematic analysis within an essentialist/realist framework, as described by Braun and Clarke. This approach was chosen to identify, analyze, and report patterns (themes) within the data at a semantic level, capturing the explicit meanings communicated by the participants. To ensure the rigor and credibility of the analysis, several strategies were employed. Investigator triangulation was achieved through independent coding and constant discussion between the two primary analysts to challenge interpretations and reach consensus. An audit trail of analytical decisions was maintained. Regarding reflexivity, both analysts are physicians (one a pediatric neurologist, one a medical education researcher) familiar with the local training context. They engaged in bracketing discussions prior to analysis to explicitly acknowledge their preconceptions about residency selection. During analysis, they maintained reflective memos to monitor how their positions might influence interpretation, striving to foreground the participants’ voices. These measures aimed to enhance the confirmability (neutrality) and dependability (consistency) of the findings. The final themes are presented in the Results alongside representative quotations. All analyses were performed using Statistical Package for the Social Sciences (SPSS) version 27 (IBM Corp., Armonk, NY) or equivalent statistical software.

### Outcomes

The primary outcome of the study was to determine the relative importance assigned to different candidate attributes, specifically comparing academic credentials, non-cognitive qualities, and research or leadership experience. Secondary outcomes included identifying variability in perceptions based on PD demographics and synthesizing qualitative themes describing the characteristics of the “ideal candidate.”

## Results

### Participant demographics

A total of 43 physicians participated in the study, comprising 9 current PDs (90% response rate among accessible PDs, representing 75% of all 12 accredited PD positions) and 34 non-PDs. The demographic and professional characteristics of the respondents are summarized in Table 1. The cohort had a mean age of 44.4 years (± 9.5) with a majority of male respondents (58.1%). Most practiced in Saudi Arabia or the UAE (95.3%), which corresponds to the locations of the SCFHS-accredited programs. One participant (2.3%) practiced in another country. A significant difference was observed in training background, with a higher proportion of current PDs having completed their residency or fellowship in North America or Europe compared to non-PDs (66.7% vs. 44.1%, *p* = 0.03). No other significant demographic differences were found between the two groups.

**Table 1:** Demographic and professional characteristics of respondents

Characteristic	Total (*N* = 43)	Current PDs (*n* = 9)	Non-PDs (*n* = 34)	*p*-value
Age (Years), Mean (SD)	44.4 (9.5)	42.3 (7.5)	44.9 (10.0)	0.46
Gender, n (%)				0.73
Male	25 (58.1%)	5 (55.6%)	20 (58.8%)	
Female	18 (41.9%)	4 (44.4%)	14 (41.2%)	
Country of Practice, n (%)				0.09
Saudi Arabia	36 (83.7%)	7 (77.8%)	29 (85.3%)	
UAE / Other	7 (16.3%)	2 (22.2%)	5 (14.7%)	
Institution Type, n (%)				0.21
Government Hospital	20 (46.5%)	3 (33.3%)	17 (50.0%)	
University/Academic Hospital	15 (34.9%)	4 (44.4%)	11 (32.4%)	
Specialized/Private Center	8 (18.6%)	2 (22.2%)	6 (17.6%)	
Training Country, n (%)				**0.03**
Saudi Arabia	22 (51.2%)	3 (33.3%)	19 (55.9%)	
North America/Europe	21 (48.8%)	6 (66.7%)	15 (44.1%)	
Specialty Background, n (%)				0.09
Pediatric Neurology	38 (88.4%)	9 (100%)	29 (85.3%)	
Pediatrics	5 (11.6%)	0 (0%)	5 (14.7%)	

### Prioritization of candidate attributes

When asked to identify the single most important factor for candidate acceptance, thematic analysis of free-text responses revealed a strong consensus across all respondents, with professionalism and attitude being the predominant choice, followed by clinical rotation performance (Table 2).

**Table 2:** Prioritization of selection criteria: qualitative ranking and quantitative importance scores

Attribute	Cited as “Most Important” Factor, *n* (%)	Mean Importance Score (1–5) ± SD	*p*-value (PD vs. Non-PD)
Professionalism & Attitude	30 (69.8%)	PD: 5.0 ± 0.0	0.16
		Non-PD: 4.9 ± 0.4	
Communication Skills	15 (34.9%)	PD: 5.0 ± 0.0	0.03
		Non-PD: 4.5 ± 0.9	
Clinical Rotation Performance	25 (58.1%)	PD: 4.8 ± 0.4	0.17
		Non-PD: 4.4 ± 1.0	
Interview Performance	6 (14.0%)	PD: 4.7 ± 0.5	0.04
		Non-PD: 3.9 ± 1.2	
Academic Performance (GPA)	9 (20.9%)	PD: 4.2 ± 0.8	0.08
		Non-PD: 3.6 ± 1.0	
Research Experience	4 (9.3%)	PD: 3.6 ± 0.9	0.25
		Non-PD: 3.2 ± 1.0	
Letters of Recommendation	5 (11.6%)	PD: 3.3 ± 0.9	0.95
		Non-PD: 3.3 ± 1.2	
Academic Performance (SMLE)	2 (4.7%)	PD: 3.3 ± 1.5	0.49
		Non-PD: 3.0 ± 1.0	

Theme 1: foundational character attributes were consistent across both PDs and non-PDs; responses emphasized intrinsic personal qualities. The most frequently cited attributes were commitment to the specialty, intellectual curiosity, and resilience. One PD described the ideal candidate as “someone who values these precious years of training as an opportunity to grow both clinically and personally.” Another emphasized “patience and meticulous observation, consistent reading and learning, and the ability to work under pressure effectively.” Non-PDs similarly highlighted “eagerness to learn, openness to change, and commitment” as essential.

Theme 2: Relational and communication competencies, where communication skills emerged not merely as a technical ability but as a reflection of deeper professional identity. Respondents described the ideal candidate as “a good communicator,” “empathetic and humble,” and possessing “strong teamwork skills, knowing when to collaborate effectively within a group and when to take initiative and lead with confidence and integrity.” PDs particularly emphasized communication as foundational to patient care and team function, with one noting the importance of “professional and personal integrity, growth mindset, and self-directed learning.”

Theme 3: Demonstrated professional behaviors where PDs valued as tangible evidence of professionalism through observable behaviors. This included “punctuality, resilience, and tolerance,” “respect, care, and commitment,” and the ability to demonstrate “self-confidence and decision-making skills.” Several respondents emphasized that ideal candidates show “consistent growth and improvement over time, both in their clinical skills and professional maturity.” One PD specifically noted the importance of “demonstrated interest with supporting documents,” suggesting that words must be accompanied by actions.

These qualitative findings align with the quantitative prioritization of professionalism and communication while revealing the nuanced ways respondents conceptualize these attributes in the context of pediatric neurology training. The convergence of themes across PDs and non-PDs reinforces the community-wide commitment to holistic candidate evaluation.

Quantitative ratings confirmed these priorities, with both PDs and non-PDs assigning the highest mean importance scores to these non-cognitive and clinical attributes. However, significant differences emerged between the groups: PDs rated communication skills significantly higher than non-PDs (mean score 5.0 vs. 4.5, *p* = 0.03) and also placed greater importance on interview performance (4.7 vs. 3.9, *p* = 0.04). In contrast, there were no significant differences in the ratings for academic metrics such as GPA, SMLE scores, or research experience.

### Perceptions on research and clinical training

In evaluating candidates’ scholarly potential, respondents most valued first-author publications (51.2%) and conference presentations (46.5%) as evidence of meaningful research engagement. To determine if the higher valuation of attributes by PDs was confounded by their greater likelihood of international training, a logistic regression was performed. This confirmed that higher importance placed on communication skills (aOR = 5.81, *p* = 0.03) and interview performance (aOR = 2.45, *p* = 0.045) were independently associated with the PD role when controlling for GPA importance and training location (Table 3).

**Table 3:** Multivariable logistic regression for PD predictors

Predictor	Adjusted Odds Ratio (aOR)	95% CI for aOR	*p*-value
Communication Skills Importance	5.81	1.18–28.6	
Interview Performance Importance	2.45	1.02–5.88	0.045
GPA Importance	1.65	0.72–3.79	0.24
Trained Abroad	2.10	0.38–11.5	0.39

## Discussion

This study offers the first structured evaluation of how pediatric neurology PDs in our region prioritize attributes when selecting residency candidates. The findings reveal a consistent emphasis on professionalism, attitude, and communication; attributes that increasingly shape contemporary residency recruitment worldwide [9]. This signals a shift from reliance on traditional academic metrics toward a more holistic appreciation of behavioral competencies that predict clinical success.

Interpretation of main findings.

The prominence of professionalism and attitude, reported as the top criterion by nearly 70% of participants, reflects the nature of pediatric neurology practice. The specialty requires sustained communication with families, coordination across multidisciplinary teams, and management of emotionally complex clinical scenarios. Consequently, directors favored observed clinical behaviors, particularly during clinical rotations, over board scores or GPA. These findings mirror literature from North America and Europe showing that real-world clinical performance and interpersonal competence better predict residency success than standardized testing alone [11, 13].

Communication skills were valued particularly highly by PDs, likely because they shoulder responsibility for resident progression and often witness firsthand how communication challenges impact patient care, team dynamics, and clinical safety. Similar emphasis has been noted in pediatric residency selection internationally, where structured interviews and behavioral assessments are increasingly adopted to evaluate empathy, clarity, and adaptability [8, 9]. This distinction is functionally significant: the gatekeepers of the specialty uniquely prioritize the interpersonal and evaluative skills essential for both candidate assessment and clinical leadership. For applicants, excelling in these areas is therefore strategically critical.

### Comparison with local and international evidence

At the national level, these findings align with growing Saudi literature advocating for holistic selection in residency programs. Prior studies emphasize balancing cognitive performance with interpersonal attributes, a trend reinforced in SCFHS policy and interview structures. Our results extend this evidence specifically to pediatric neurology and suggest that directors in this subspecialty apply the same principles seen in broader medical training (Table 4).

**Table 4:** Benchmarking with global literature

Study	Country	Specialty	Top Traits Identified
Warm et al., 2022 [9]	United States	Pediatrics	Communication, adaptability
AlAssiri et al., 2024 [8]	Saudi Arabia	Surgery	Professionalism, teamwork
Our findings	Saudi Arabia	Pediatric Neurology	Professionalism, communication, interview performance

Internationally, the trend toward competency-based selection is evident in the U.S. shift to pass/fail Step 1 scoring and the adoption of frameworks like CanMEDS in Canada [1]. The convergence of Saudi practices with these models suggests a shared commitment to selecting clinicians who are not only knowledgeable but also dependable, collaborative, and patient-centered.

### Implications for training and policy

The prioritization of professionalism and communication has clear implications for training programs and policy development. Residency programs may benefit from embedding structured behavioral interviews, situational judgment tests, or validated professionalism tools into their selection processes. For SCFHS, the findings support refining recruitment guidelines to more explicitly integrate non-cognitive domains into evaluation criteria.

Medical schools also have a role: early mentorship, longitudinal professionalism curricula, and opportunities for meaningful clinical exposure may better prepare students for specialties such as pediatric neurology, where interpersonal attributes strongly influence success [14, 15].

### Strengths, limitations, and future directions

A major strength of this study is its national scope within a small subspecialty, incorporating perspectives from both PDs and non-PDs. However, the sample size reflects the limited number of pediatric neurology programs, and perceptions may not perfectly mirror actual ranking behavior. Future research should examine whether the attributes valued by directors correlate with long-term resident performance and patient-centered outcomes. It also should incorporate resident and applicant perspectives for a broader view. Furthermore, future studies are recommended to evaluate the impact of these selection priorities on educational outcomes and to explore the broader research landscape in pediatric neurology training.

## Conclusion

Pediatric neurology PDs in Saudi Arabia and the UAE place substantial weight on professionalism, communication, and observed clinical performance, aligning with global trends toward competency-based and humanistic residency selection. While academic metrics remain important, they appear secondary to behavioral attributes that predict resilience, teamwork, and compassionate care. These findings highlight opportunities to strengthen national selection frameworks, enrich training pathways, and ultimately cultivate a pediatric neurology workforce equipped to meet the complex needs of children and families across the region.

## Supplementary Information

Supplementary Material 1.

## Data Availability

The datasets generated and analyzed during the current study are not publicly available to protect the anonymity and confidentiality of the participants but are available from the corresponding author on reasonable request.

## Abbreviations

ADHD: Attention–Deficit/Hyperactivity Disorder
aOR: Adjusted Odds Ratio
CI: Confidence Interval
EEG: Electroencephalogram
EMG: Electromyogram
GPA: Grade Point Average
IBM: International Business Machines
IRB: Institutional Review Board
KAU: King Abdulaziz University
KAUH: King Abdulaziz University Hospital
KFSHRC: King Faisal Specialist Hospital & Research Centre
Likert scale: Likert–type rating scale
MOH: Ministry of Health
Non: PDs–Non–Program Directors
PD: Program Director
PDs: Program Directors
SCFHS: Saudi Commission for Health Specialties
SD: Standard Deviation
SMLE: Saudi Medical Licensure Examination
SPSS: Statistical Package for the Social Sciences
STROBE: Strengthening the Reporting of Observational Studies in Epidemiology
UAE: United Arab Emirates
